# OLA-1, an Obg-like ATPase, integrates hunger with temperature information in sensory neurons in *C. elegans*

**DOI:** 10.1371/journal.pgen.1010219

**Published:** 2022-06-08

**Authors:** Ichiro Aoki, Paola Jurado, Kanji Nawa, Rumi Kondo, Riku Yamashiro, Hironori J. Matsuyama, Isidre Ferrer, Shunji Nakano, Ikue Mori

**Affiliations:** 1 Group of Molecular Neurobiology, Neuroscience Institute, Graduate School of Science, Nagoya University, Nagoya, Japan; 2 Division of Biological Science, Graduate School of Science, Nagoya University, Nagoya, Japan; 3 Cancer Area, Institut d’Investigació Biomèdica de Bellvitge, Barcelona, Spain; 4 Neuroscience Area, Institut d’Investigació Biomèdica de Bellvitge, Barcelona, Spain; Brown University, UNITED STATES

## Abstract

Animals detect changes in both their environment and their internal state and modify their behavior accordingly. Yet, it remains largely to be clarified how information of environment and internal state is integrated and how such integrated information modifies behavior. Well-fed *C*. *elegans* migrates to past cultivation temperature on a thermal gradient, which is disrupted when animals are starved. We recently reported that the neuronal activities synchronize between a thermosensory neuron AFD and an interneuron AIY, which is directly downstream of AFD, in well-fed animals, while this synchrony is disrupted in starved animals. However, it remained to be determined whether the disruption of the synchrony is derived from modulation of the transmitter release from AFD or from the modification of reception or signal transduction in AIY. By performing forward genetics on a transition of thermotaxis behavior along starvation, we revealed that OLA-1, an Obg-like ATPase, functions in AFD to promote disruption of AFD-AIY synchrony and behavioral transition. Our results suggest that the information of hunger is delivered to the AFD thermosensory neuron and gates transmitter release from AFD to disrupt thermotaxis, thereby shedding light onto a mechanism for the integration of environmental and internal state to modulate behavior.

## Author summary

As we humans perceive food smell more attractive when we are hungry, animal’s internal state such as satiety affects their sensory stimulus-induced behavior. However, it is not fully understood how multiple external and internal inputs are integrated in the nervous system to modify behavior. In this study, we analyzed the effect of starvation on the thermotaxis behavior of the nematode *C*. *elegans*. Animals migrate toward past cultivation temperature on a thermal gradient without food, which is disrupted by starvation. We have found that an ATPase, called OLA-1, which is universally conserved both in prokaryotes and eukaryotes, acts in the thermosensory neuron to modulate its communication with a downstream interneuron, resulting in a modification of the thermotaxis behavior. Our results provided a molecular and neural-circuit mechanism by which animals integrate information of their internal state with that of the external environment to modify their behavior.

## Introduction

Animals detect changes in the environment and their internal state and modify their behavior accordingly to survive a heterogenous world. However, it remains largely to be clarified how information of environment and internal state is integrated and how this integrated information modifies behavior. Satiety-hunger axis is a crucial aspect of internal state, and starvation indeed modifies a wide variety of sensory behaviors both in vertebrates [1,2] and invertebrates [3–5]. In *C*. *elegans*, starvation enhances feeding [6], decelerates locomotion on food lawn [7,8], prompts males to prioritize feeding over exploration in search for hermaphrodites [9,10], drives male mating less efficient [11], converts response to CO_2_ from aversion to attraction [12–14], modifies O_2_-response [15], enhances avoidance of pheromone [16] and disrupts chemotaxis to odorants [17–21] and NaCl [22–24].

Thermotaxis of *C*. *elegans* is also disrupted by starvation [25–31]. *C*. *elegans* associates cultivation temperature with food existence and migrates toward the past cultivation temperature on a thermal gradient without food to search for food and track isothermally [25]. Previous works have revealed neurons involved in this thermotaxis [32–35]. Within the neural circuit, AFD is the major thermosensory neuron, which is activated by warming [36–43]. The lower threshold temperature for AFD activation is dependent on the past cultivation temperature. AFD forms chemical synapses predominantly onto AIY interneuron [44,45], which is also essential for thermotaxis [32,46]. We and another group recently reported that AIY is activated synchronously with AFD when animals are below cultivation temperature [31,47,48], which drives thermotaxis toward higher temperature up a thermal gradient. When starved, animals do not migrate to the cultivation temperature. In starved animals, AFD activity is unaltered [27,30,31,49], while the synchrony between AFD and AIY is disrupted [31]. However, it is still unknown whether the disruption of AFD-AIY synchrony is caused by altered release of transmitters from AFD or by altered reception or signaling in AIY.

**Fig 1:**
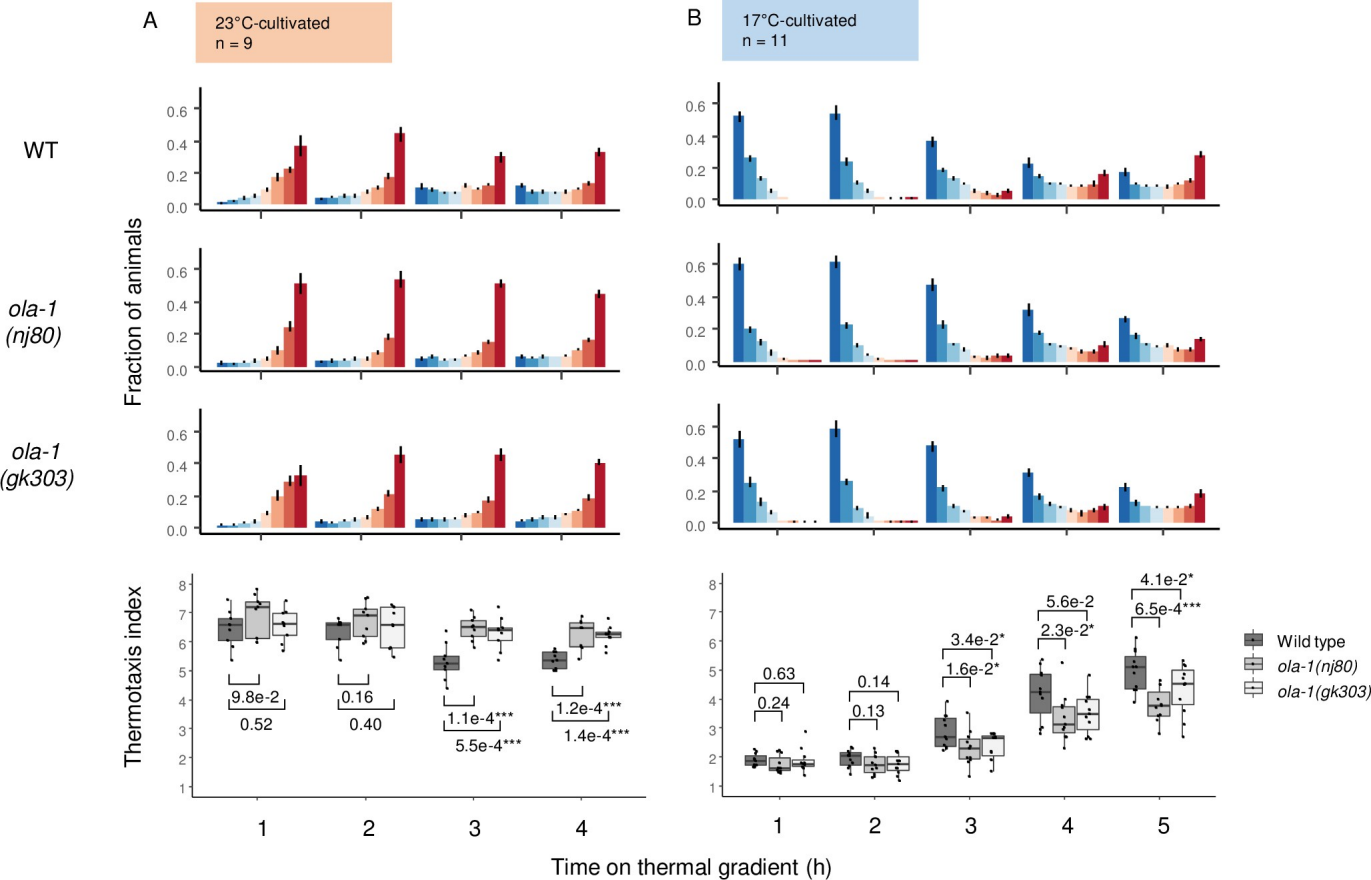
*ola-1* mutants are slower in the transition of the thermotactic behavior. Wild type, *ola-1(nj80)* and *ola-1(gk303)* animals were cultivated at 23°C for 3 days (A) or at 17°C for 5 days (B) and allowed to freely migrate on a thermal gradient for the time indicated. Number of animals at each section of the plate was scored. Fraction of animals (upper) and thermotaxis indices (lower) are shown. p values were indicated (one-tailed Dunnett test against wild type animals at each time point).

Here, we identified OLA-1, an Obg-like ATPase, as a molecule that functions in AFD to disrupt AFD-AIY synchrony in starved animals and to promote transition from isothermal tracking (IT) to dispersion. An *ola-1* allele was isolated from a forward genetic screen for mutants that were slower than wild type to start dispersing from the cultivation temperature when left longer on a thermal gradient without food (Fig 1). OLA-1 acted in AFD thermosensory neurons when cultivated at a relatively high temperature such as 23°C but in multiple neurons when cultivated at a relatively low temperature such as 17°C. However, AFD calcium response was similar between wild type and *ola-1* mutant animals that were cultivated at 23°C and tracking isothermally on a thermal gradient, whereas *ola-1* was required for the disruption of the AFD-AIY synchrony after starvation. In addition, we found that ZYG-8, which is homologous to mammalian doublecortin-like kinase (DCLK) [50] and interacts with OLA-1 [51], decelerated the transition from IT to dispersion. Taken together, our findings suggest that the information about hunger is delivered to AFD and gates the output from AFD to regulate the transition from IT to dispersion during the disruption of thermotaxis.

## Results

### *ola-1* is slow in altering behavior on a thermal gradient

Well-fed animals migrate to the past cultivation temperature on a thermal gradient, while starved animals do not. To observe how starvation affects the thermotaxis more in detail, we monitored the behavior of *C*. *elegans* on a thermal gradient for a long period (Figs 1 and S1). When wild type animals cultivated either at 23°C or 17°C were allowed to migrate freely on a thermal gradient for one hour, the center of which was set to 20°C, they accumulated at the temperature of previous cultivation, indicating isothermal tracking (IT) as characterized previously [25,32,35,52,53]. However, when left longer on the thermal gradient, animals started dispersing in several hours and dispersed almost evenly within 24 hours regardless of their previous cultivation temperature (Figs 1 and S1A and S1B). From a forward genetic screen (S1C Fig), *nj80* was isolated as a mutant allele that remained longer at the past cultivation temperature (Figs 1 and S1).

**Fig 2:**
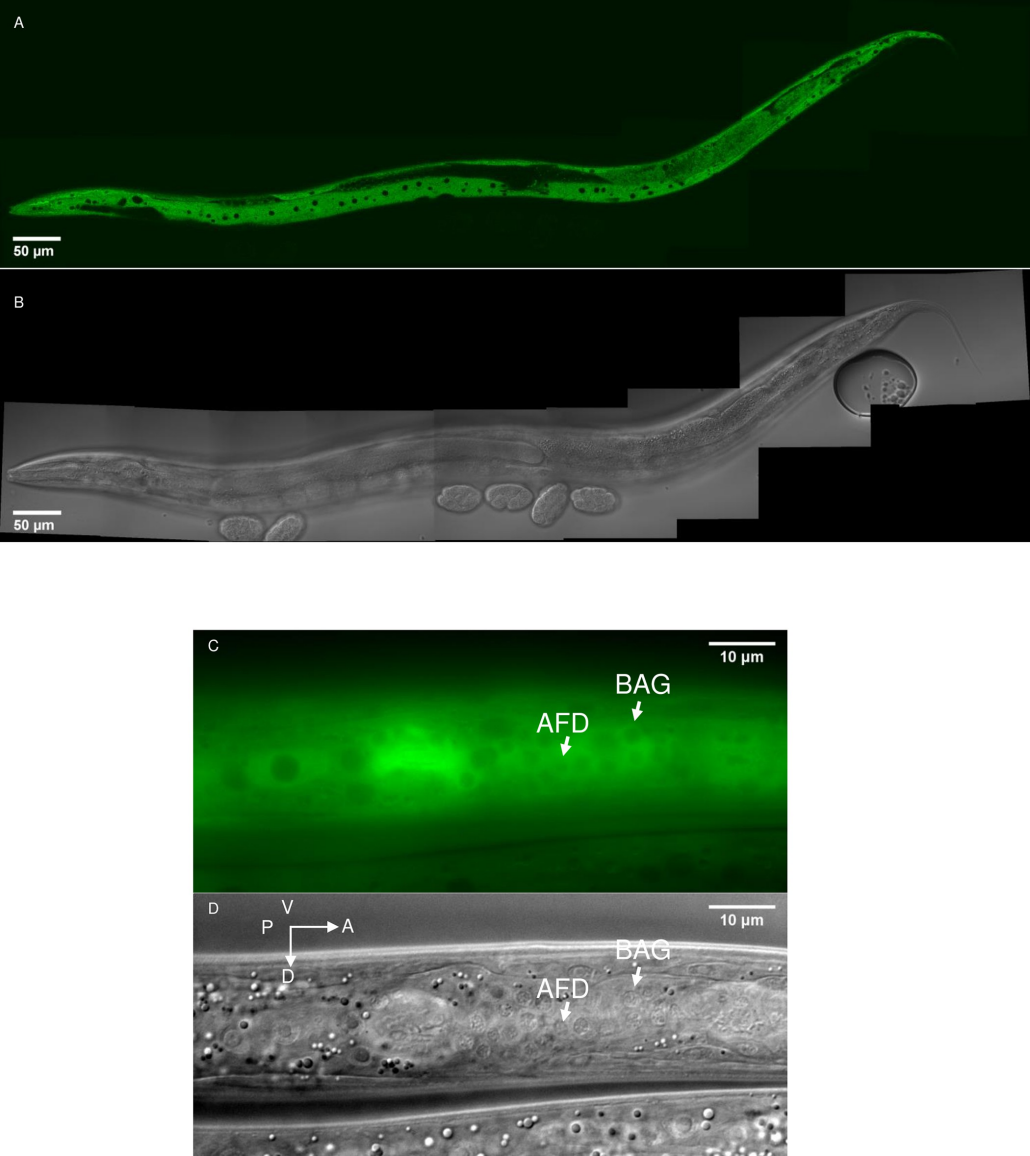
*ola-1* expression. *ola-1(nj80); njEx1668[ola-1p*::*ola-1*::*GFP]* animals were subjected to microscopic analysis. GFP fluorescence and DIC images for the whole-body (A) and head region (B).

**Fig 3:**
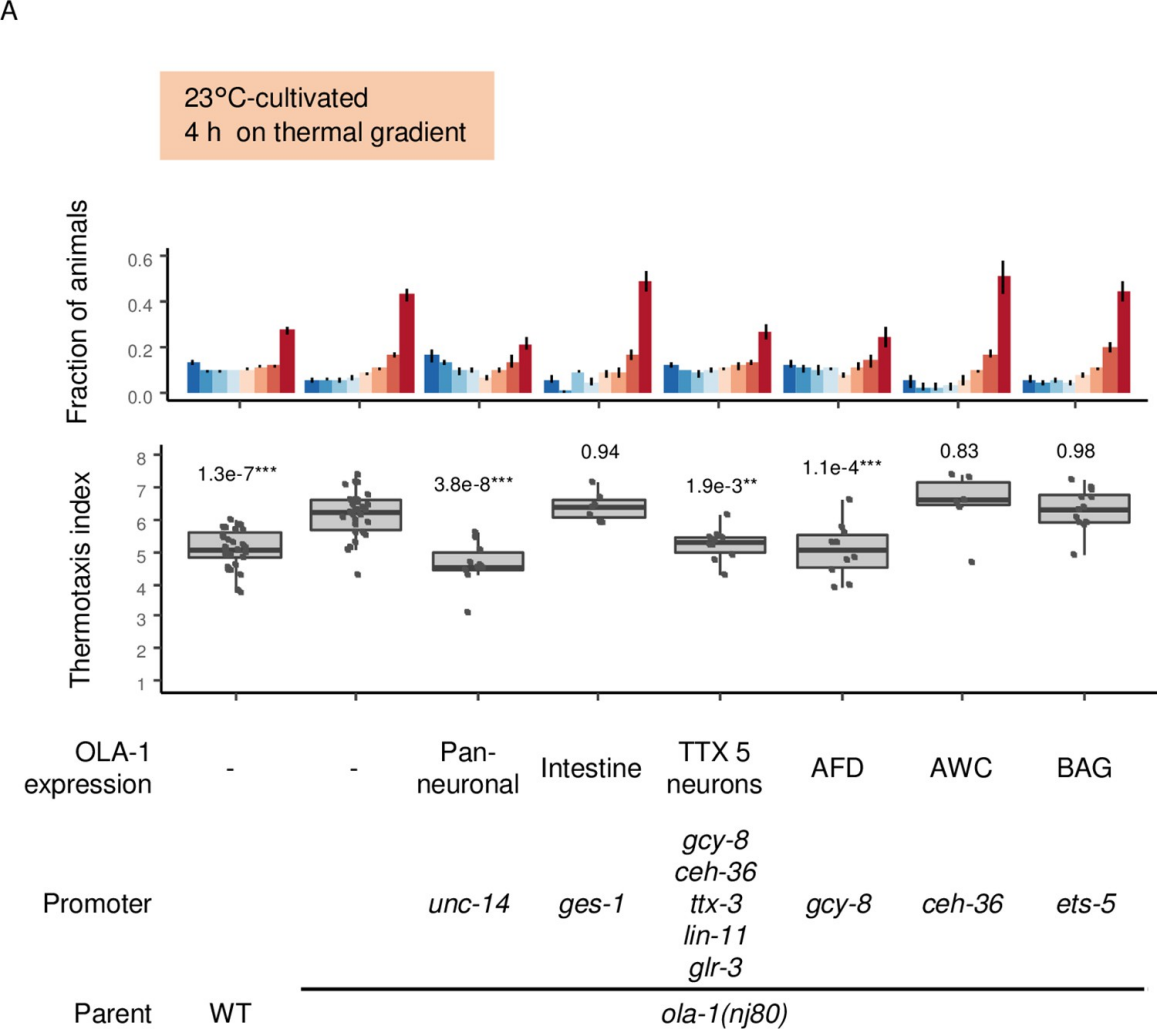
*ola-1* acts in AFD thermosensory neurons when animals are cultivated at 23°C. Wild type and *ola-1(nj80)* animals and *ola-1(nj80)* animals that express OLA-1 in cells or tissues indicated were cultivated at 23°C for 3 days and allowed to freely migrate on a thermal gradient for 4 h. n = 30, 30, 10, 7, 10, 10, 6, 12. p values are indicated (Dunnett test against *ola-1(nj80)* animals).

**Fig 4:**
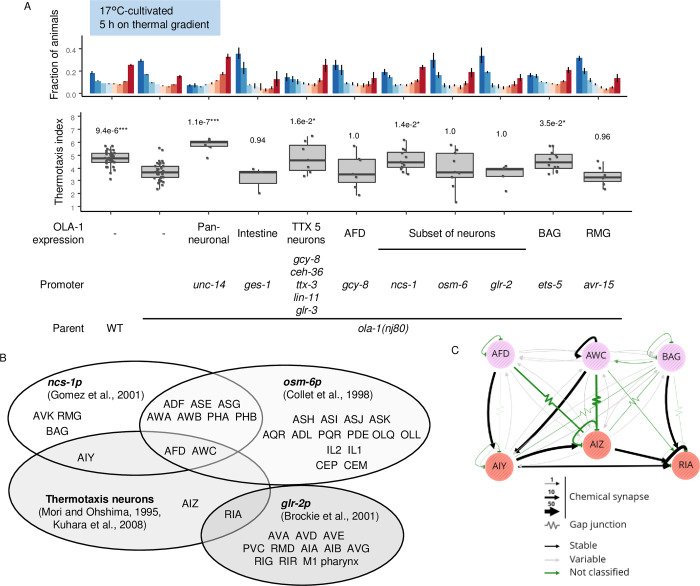
*ola-1* acts in multiple neurons when animals are cultivated at 17°C. (A) Wild type and *ola-1(nj80)* animals and *ola-1(nj80)* animals that express OLA-1 in the cells or tissues indicated were cultivated at 17°C for 5 days and allowed to freely migrate on a thermal gradient for 5 h. n = 36, 37, 7, 3, 7, 7, 12, 9, 5, 12, 7. p values are indicated (Dunnett test against *ola-1(nj80)* animals). (B) Neurons where each gene promoter induces gene expression. (C) Connections of indicated neurons were visualized by NemaNode (https://bit.ly/3BSnHAj) [45].

By SNP mapping and whole-genome sequencing, *nj80* was mapped to a mutation at a splicing acceptor site of the second intron of the *ola-1* gene, which encodes an Obg-like ATPase [54]. A deletion allele of *ola-1*, *gk303*, was also slower than wild type in dispersing from cultivation temperature on a thermal gradient (Fig 1), supporting that the *nj80* is an *ola-1* allele. When OLA-1 was fused to GFP and expressed under *ola-1* promoter, GFP fluorescence was observed in the nervous system, pharyngeal muscles and intestine (Fig 2A and 2B). Pan-neuronal expression of OLA-1 rescued abnormality of *ola-1* mutants but that in intestine did not (Figs 3 and 4A), indicating that the loss of *ola-1* function causes the slow dispersion and that OLA-1 functions in the nervous system to accelerate transition from IT to dispersion.

*ola-1(nj80)* showed slightly larger thermotaxis index when animals were cultivated at 23°C and left on a thermal gradient for 1 hour (S1D Fig), which could be due to rigid preference for 23°C since *ola-1(nj80)* did not prefer higher temperature (S1E Fig). Given that *ola-1(gk303)*, a different allele of *ola-1*, did not rigidly prefer 23°C after 1 hour on a thermal gradient but still showed slower dispersion (Fig 1A), the rigid preference for 23°C, as observed in *ola-1(nj80)*, does not seem to cause the slow dispersion.

### OLA-1 acts in different sensory neurons depending on the cultivation temperature

To understand how OLA-1 regulates the transition from IT to dispersion, we aimed to determine in which neurons OLA-1 acts. When animals were cultivated at 23°C, OLA-1 expression in five pairs of neurons (AFD, AWC, AIY, AIZ and RIA) previously shown to be involved in thermotaxis [32,33] rescued abnormality of *ola-1* mutants (Fig 3). Of these five pairs of neurons, OLA-1 expression in AFD thermosensory neurons rescued abnormality of *ola-1* mutants but expression in AWC chemo/thermosensory neurons did not (Fig 3). These results suggest that OLA-1 acts in AFD to accelerate transition from IT to dispersion when animals are cultivated at 23°C.

When animals were cultivated at 17°C, pan-neural OLA-1 expression and expression in thermotaxis-related five pairs of neurons rescued the abnormality of *ola-1* mutants, whereas AFD-specific OLA-1 expression did not (Fig 4A). We also expressed OLA-1 in different subsets of neurons by fusing *ola-1* cDNA with a set of promoters (Fig 4A and 4B). OLA-1 expression by *ncs-1* promoter [55] rescued abnormality of *ola-1* mutant animals, whereas expression by *osm-6* [56] or *glr-2* [57] promoters did not. OLA-1 expression by *ets-5* promoter, which is active in BAG sensory neuron [58], also rescued *ola-1* abnormality (Fig 4A). These results suggest that OLA-1 can function in multiple neurons including thermotaxis-related neurons and BAG neurons when animals are cultivated at 17°C. OLA-1 expression by *ets-5* promoter did not rescue abnormality of *ola-1* mutants cultivated at 23°C (Fig 3). Since the abnormality of *ola-1* mutants and the rescue effect were more robust when animals were cultivated at 23°C, we mainly performed experiments under this condition hereafter.

*ola-1* mutants grew slowly both at 23°C and 17°C, and these defects were not rescued by OLA-1 expression under *gcy-8* and *ets-5* promoters, respectively (S2 Fig), which had rescued the slower dispersion (Figs 3 and 4). These results indicate that the slower dispersion observed in *ola-1* mutants is not caused by a general slowdown of the animals’ biological clock.

### *ola-1* is defective for disruption of thermotaxis after starvation

**Fig 5:**
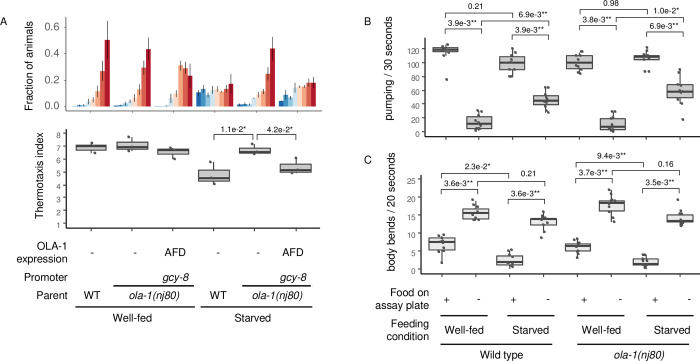
*ola-1* does not modify thermotaxis behavior after starvation. (A) Wild type and *ola-1(nj80)* animals were cultivated at 23°C, transferred to NGM plates with (well-fed) or without (starved) food and cultivated at 23°C for 2 h. Animals were then allowed to freely migrate on a thermal gradient for 1 h. n = 3. p values are indicated (one-tailed Dunnett test against starved *ola-1(nj80)* animals). (B-C) Well-fed and starved animals of wild type and *ola-1(nj80)* were transferred to new NGM plates with or without bacterial food and recorded. Pharyngeal pumping (B) or body bending (C) were visually counted. n = 10. p values were indicated (Steel-Dwass test).

We previously reported that starved *C*. *elegans* no longer migrate to the cultivation temperature [26–29]. Since *ola-1* mutants are possibly defective in integrating hunger information when staying longer around the cultivation temperature on a thermal gradient (Fig 1), we examined whether *ola-1* mutants migrate toward cultivation temperature when starved before being transferred to a thermal gradient. Wild type animals starved at 23°C dispersed on a thermal gradient, whereas starved *ola-1* mutants migrated to the 23°C region to the same extent as non-starved animals. This defect of *ola-1* mutants was rescued by AFD-specific *ola-1* expression (Fig 5A). These results suggest that OLA-1 acts in AFD to disrupt thermotaxis behavior after starvation.

Since starvation did not disrupt thermotaxis of *ola-1* mutants, we next asked whether ola-1 can sense the presence/absence of food and hunger. *C*. *elegans* feeds through pharyngeal pumping, which is affected by the presence of bacterial food and starvation [6]. Both well-fed and starved wild type animals pumped much slower in the absence of food, and the starvation accelerated pumping in the absence of food (Fig 5B), consistently with the previous report [6]. This was also the case in ola-1 mutants, indicating that *ola-1* mutants can sense food and starvation.

Locomotion rate of *C*. *elegans* is also affected by the presence of bacterial food and starvation [7]. As described previously, wild type animals slowed down when they entered bacterial lawn, which was more pronounced in the starved than the well-fed animals (Fig 5C). This was again also the case in *ola-1* mutants, further confirming that *ola-1* mutants can sense food and starvation. Taken together, *ola-1* mutants seem to be specifically defective in integrating the sense of hunger to alter thermotaxis behavior.

### OLA-1 functions downstream of Ca^2+^ increase in AFD to modify AFD-AIY synchrony under starvation

**Fig 6:**
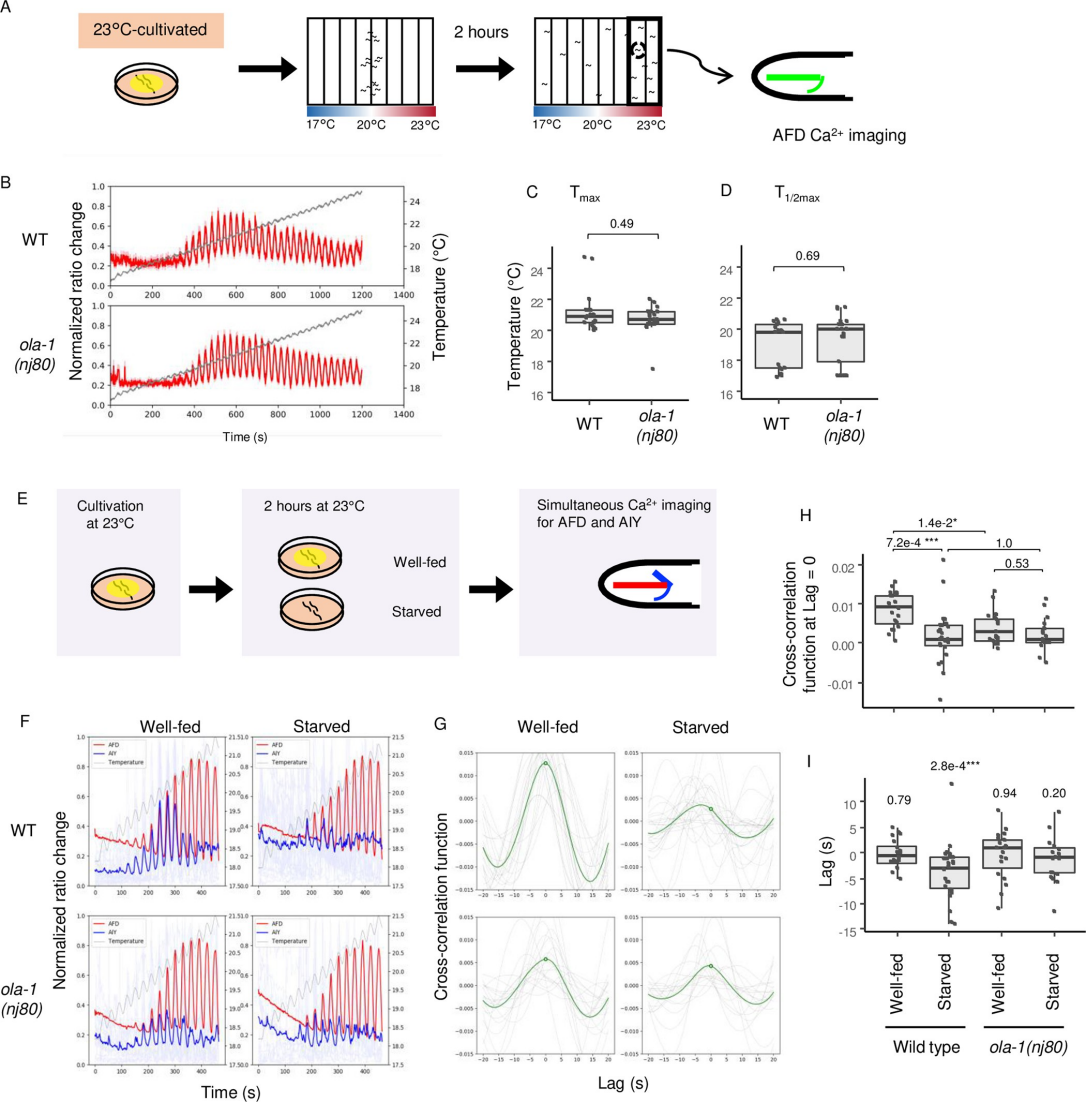
OLA-1 modifies synchrony between AFD and AIY after starvation. (A-D) Wild type and *ola-1(nj80)* animals expressing GCaMP3 and tagRFP in AFD were cultivated at 23°C and allowed to freely migrate on a thermal gradient for 2 hours. Animals were then collected from sections 7 and 8, immobilized and subjected to Ca^2+^ imaging analysis with the indicated temperature stimulus warming from 15°C to 24°C with oscillation (B). The ratio of green to red fluorescence of each trial was normalized from zero to one, and the mean values of normalized ratio were plotted (B). Shadow represents the SEM. Data were collected from distinct animals. Temperature at which moving average of the normalized ratio change with 5 sec of window showed the maximum (C) and the half maximum for the first time (D) were plotted. n = 17, 21. p values were indicated (Wilcoxon rank sum test). (E-I) Wild type and *ola-1(nj80)* animals expressing GCaMP3 in AIY and XCaMP-R in AFD were cultivated at 23°C, transferred to NGM plates with or without food and incubated at 23°C for 2 h. Animals were then immobilized and subjected to Ca^2+^ imaging analysis with the indicated temperature stimulus warming from 18°C to 21°C with oscillation (F). The ratio of green or red fluorescence of each trial was normalized from zero to one. The mean values of the normalized fluorescence signals of AFD and AIY were shown with solid lines in red and blue, respectively. Light-colored lines represent individual data of AIY. Dashed oscillatory curves represent mean values of the thermal stimuli (F). Data were collected from distinct animals. (G) Mean and individual values of cross-correlation function between AFD and AIY were plotted against time lag. Solid green curves indicate the mean values, and gray curves indicate the individual data. Circles indicate values of the mean cross-correlation function when Lag = 0 s. The cross-correlation function was calculated for the fluorescence signals between 101 s and 400 s. (H-I) Values of the cross-correlation function at Lag = 0 s (H) and time lag at which the cross-correlation functions take their maxima (I) of each strain at each condition were plotted. p values were indicated (Steel-Dwass test (H) and Wilcoxon signed-rank test (I)). n = 20, 25, 19, 17.

Since OLA-1 was shown to act in AFD thermosensory neurons to disrupt thermotaxis after starvation when animals were cultivated at 23°C (Figs 3 and 5), we next asked whether *ola-1* mutation affected the responsiveness of AFD. AFD increases intracellular Ca^2+^ in response to warming, and the threshold temperature from which AFD starts responding is dependent on past cultivation temperature [36,37]. Wild type and *ola-1(nj80)* animals expressing GCaMP3, a genetically encoded Ca^2+^ indicator [59], in AFD were cultivated at 23°C and allowed to freely migrate on a thermal gradient for two hours. Animals were collected from the warm region around 23°C of the thermal gradient and then subjected to Ca^2+^ imaging analysis (Fig 6A). Both in wild type and *ola-1* mutants, AFD Ca^2+^ signals similarly increased and decreased with warming and cooling, respectively, (Fig 6B–6D). This result suggests that OLA-1 may function downstream of Ca^2+^ increase in AFD to accelerate transition from IT to dispersion.

We also analyzed animals collected from cold sections 1 and 2 of the thermal gradients. Unexpectedly, AFD in *ola-1* mutants started responding from a slightly lower temperature than the wild type (S3A–S3C Fig). This might be due to the different temperature history experienced by wild type and *ola-1* mutants before arriving the low temperature region in two hours.

We next asked whether *ola-1* affects the activity of AIY interneurons that play an essential role for thermotaxis downstream of AFD [32,46]. We previously showed that AIY activities synchronize with AFD when well-fed animals are at temperature below cultivation temperature and that this synchrony is disrupted by starvation [31]. We therefore compared synchrony of AFD and AIY activities in well-fed and starved animals of wild type and *ola-1* mutant (Fig 6E–6I). AFD and AIY activities were simultaneously recorded from the same animals that express XCaMP-R [60] and GCaMP3 in AFD and AIY, respectively, under temporal thermal stimuli (Fig 6F). In wild type animals, while AFD response was not altered by starvation, the correlation between AFD and AIY decreased upon starvation (Fig 6F) as indicated by the decrease of value of the cross-correlation function at Lag = 0 (Fig 6G and 6H), in agreement with previous reports [27,31,49]. In contrast, value of the cross-correlation function at Lag = 0 did not decrease by starvation in *ola-1* mutants (Fig 6F and 6G and 6H). Moreover, time lags between AFD and AIY activities were distributed around 0 (s) in all groups except starved wild type animals (Fig 6I), consistently with the results indicating that *ola-1* is defective in disrupting thermotaxis behavior after starvation (Figs 1A and 5A). For wild type and *ola-1* mutants, Fourier power spectra of AFD calcium signal at the frequency of oscillatory thermal stimuli (0.033 Hz) did not show any significant difference between well-fed and starved animals, while those of AIY decreased by starvation in wild type animals but not in *ola-1* mutant (S3D and S3E Fig), indicating that *ola-1* mutant did not alter their AIY activities in response to starvation. Taken together, OLA-1 seems to act downstream of AFD activation to modify synchrony between AFD and AIY probably by regulating release of transmitters from AFD.

### ZYG-8 acts in AFD to decelerate the transition of behavioral strategy

**Fig 7:**
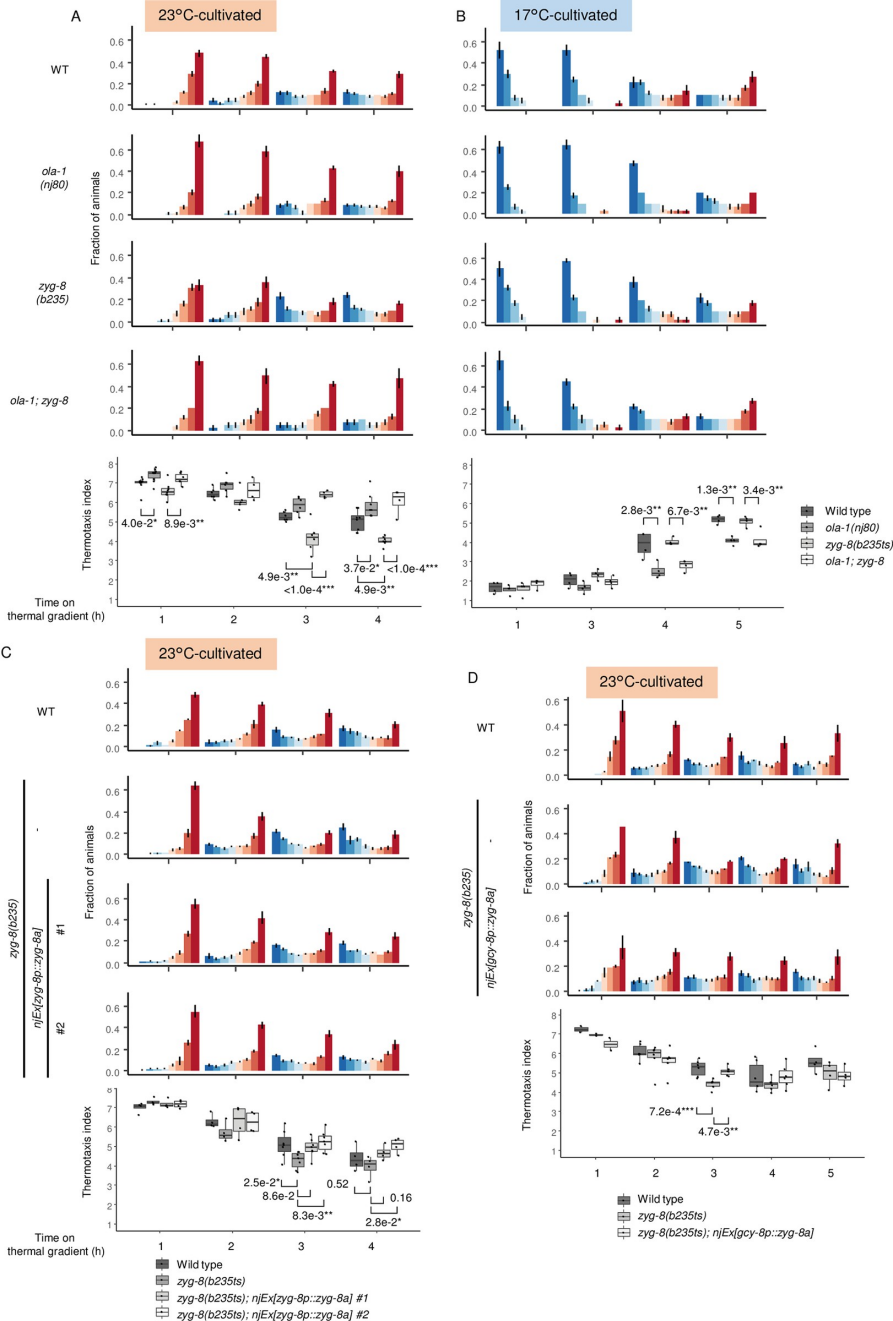
*zyg-8* mutants are faster in the transition of the thermotactic behavior. (A-B) Wild type, *ola-1(nj80)*, *zyg-8(b235ts)* and *ola-1; zyg-8* animals were cultivated at 23°C for 3 days (A) or at 17°C for 5 days (B) and allowed to freely migrate on a thermal gradient for the time indicated. p values were indicated (Tukey-Kramer test). n = 4–10 (A) and n = 4 (B). (C-D) Wild type and *zyg-8(b235ts)* animals and *zyg-8(b235ts)* animals that express ZYG-8 isoform a under *zyg-8* promoter (C) or *gcy-8* promoter (D) were cultivated at 20°C for 2.5 days and the at 23°C for 1 day to avoid severe zygotic lethality of transgenic strains. Animals were then allowed to freely migrate on a thermal gradient for the time indicated. p-values were indicated (Dunnett test against *zyg-8(b235ts)* animals). n = 4–9 (C) and n = 2–6 (D).

It was previously shown that OLA-1 physically interacts with ZYG-8, an ortholog of human doublecortin-like kinases (DCLK) [50], by an interactome study, in which *C*. *elegans* proteins were analyzed by yeast two-hybrid system [51]. We therefore examined whether ZYG-8 is also involved in the transition from IT to dispersion as is OLA-1. In contrast to *ola-1* mutants, *zyg-8(b235ts)* mutants cultivated at 23°C were faster than wild type animals to disperse from 23°C region on a thermal gradient (Fig 7A). *zyg-8(b235ts)* mutants cultivated at 17°C dispersed from 17°C region in a similar manner to wild type animals (Fig 7B). It is unclear whether *zyg-8* is dispensable for decelerating the transition of behavioral strategy when cultivated at 17°C or the point mutant (L723F for the isoform a) form of ZYG-8 in *zyg-8(b235ts)* animals is active enough to decelerate the transition, since *zyg-8(b235ts)* is a temperature sensitive allele in zygotic lethality [61]. Expression of *zyg-8a* cDNA under *zyg-8* promoter rescued the fast transition from IT to dispersion in *zyg-8(b235ts)* animals (Fig 7C), which confirmed that the behavioral defect in *zyg-8(b235ts)* animals was caused by loss of *zyg-8* function.

**Fig 8:**
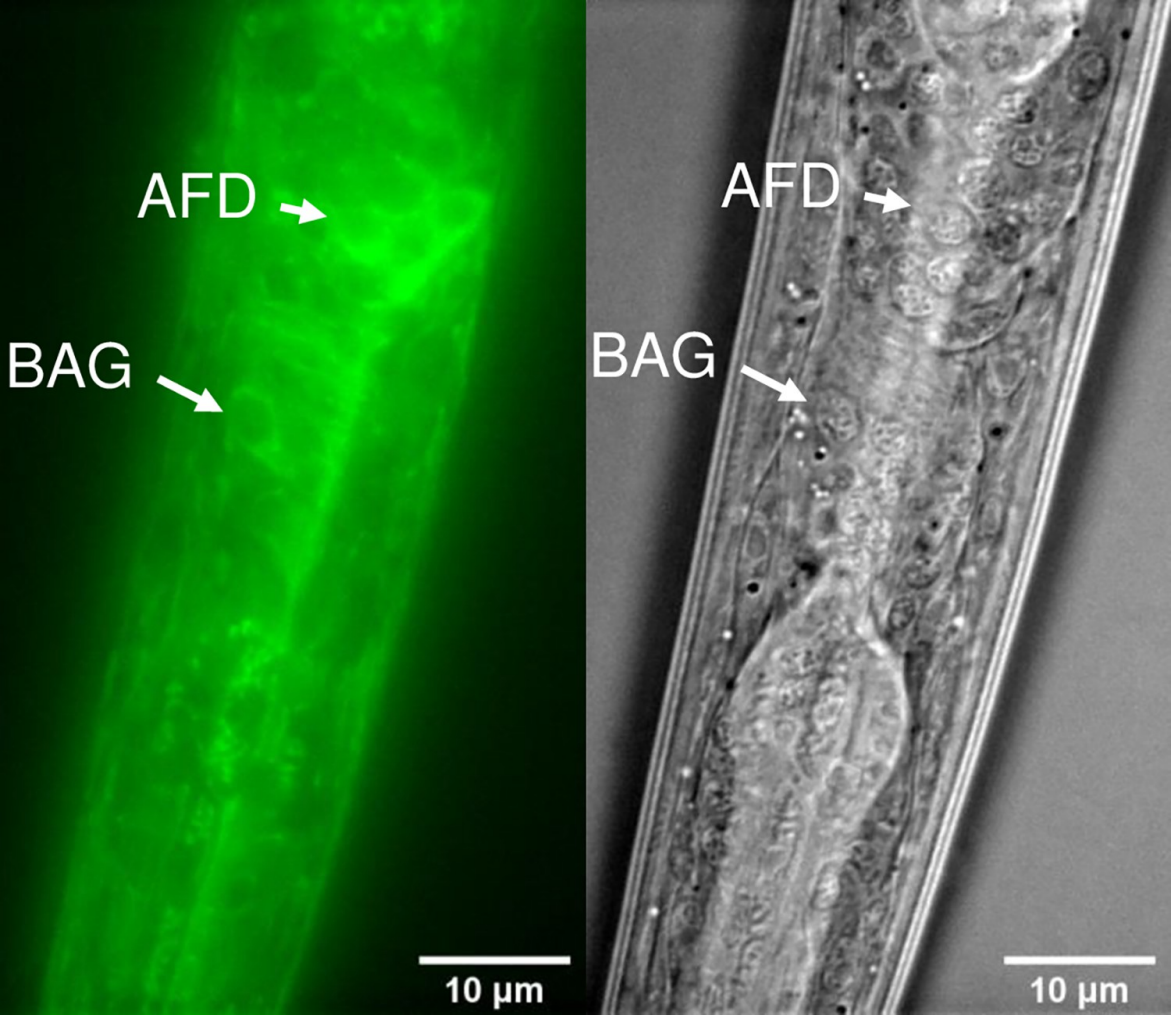
*zyg-8* expression. A *zyg-8(b235); njEx1680[zyg-8p*::*zyg-8a cDNA*::*GFP*, *ges-1p*::*tagRFP]* animal was subjected to microscopy for GFP fluorescence (left) and DIC (right) imaging.

Since the transition from IT to dispersion in *ola-1; zyg-8* double mutants were as slow as that in *ola-1* single mutants (Fig 7A), *ola-1* seemed to act downstream of or in parallel with *zyg-8*. If ZYG-8 affects the transition from IT to dispersion by physically interacting OLA-1, ZYG-8 is supposed to function in AFD in animals cultivated at 23°C, where OLA-1 functions. AFD-specific *zyg-8* expression rescued abnormality of *zyg-8* mutants (Fig 7D), indicating ZYG-8 acts in AFD. Consistently, ZYG-8 expression was observed in AFD (Fig 8).

## Discussion

In this study, we showed that OLA-1, an Obg-like ATPase, acted in AFD thermosensory neuron to promote disruption of AFD-AIY synchrony after animals were cultivated and starved at 23°C, and consequently the transition from isothermal tracking (IT) to dispersion. We had previously identified molecular and circuital mechanisms involved in the disruption of thermotaxis behavior after starvation [26–29]; insulin signaling [27] and TAX-6 calcineurin [28] acted in interneurons in this study. Our results suggested that hunger information is delivered to AFD thermosensory neurons. Contribution of AFD and interneurons are not mutually exclusive, but both could rather be involved in the behavioral transition. Note that since isothermal tracking was used in previous studies as a readout for screen, a different aspect of the behavior could have been highlighted. It was recently reported that INS-1 insulin derived from intestine modifies the activity of AWC chemo/thermosensory neuron and AIA interneuron to disrupt thermotaxis toward lower temperature down the gradient in starved animals [30]. Different mechanisms could be used for disruption of thermotaxis toward warmer and cooler temperatures, as additionally suggested from our results showing that OLA-1 did not act in AFD for the dispersion from 17°C. Feedback to sensory neurons by insulin signaling is also reported in the case of odor (AWC) [62] and salt (ASER) [23] chemotaxis. However, feedback to AFD might be mediated by something different from insulin signaling since quicker disruption of thermotaxis observed in mutants for *age-1*, which encodes phosphoinositide 3-kinase (PI3K) functioning downstream of an insulin receptor, was not rescued by *age-1* expression in AFD [27].

**Fig 9:**
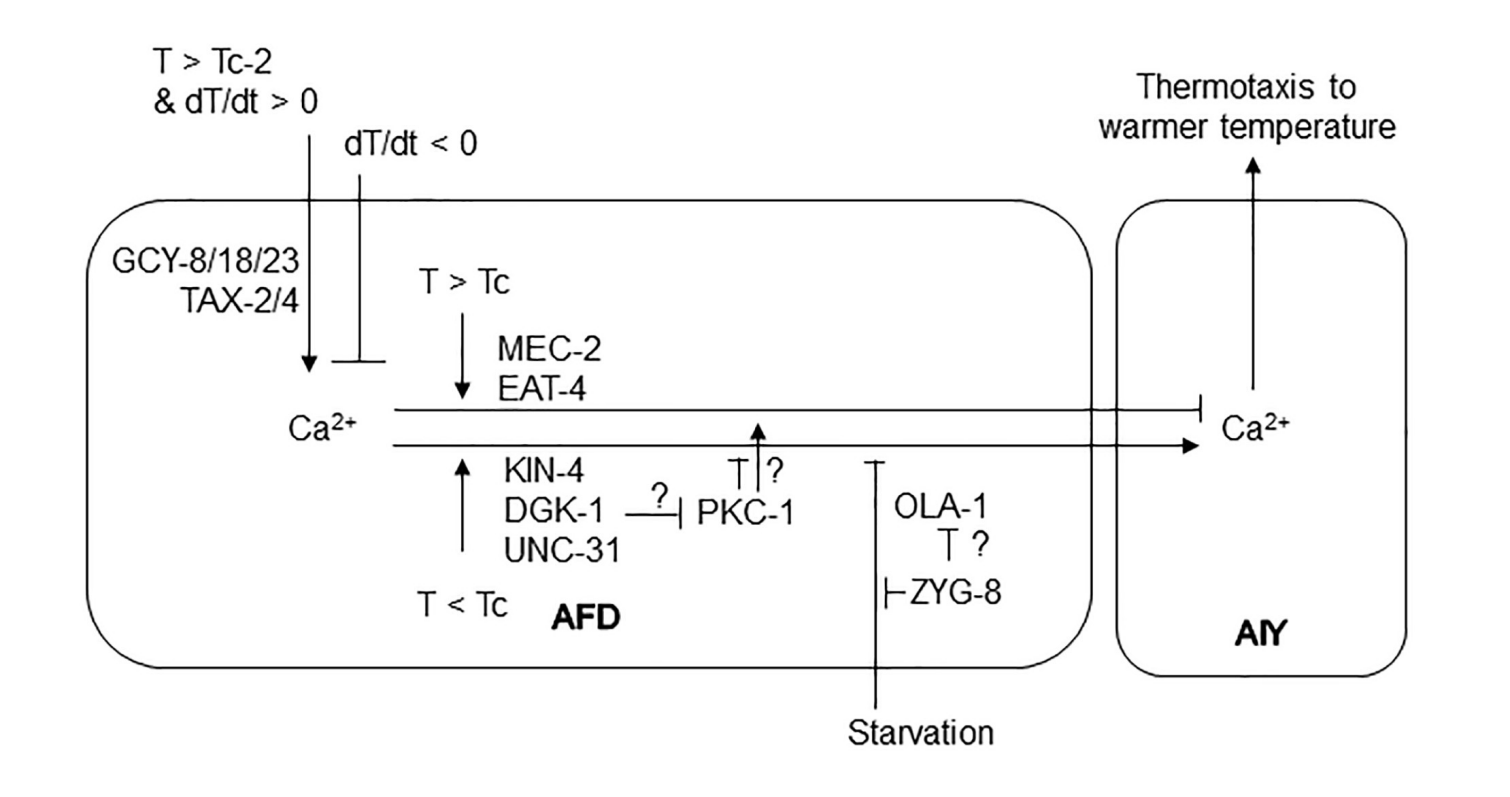
Mechanisms underlying AFD-AIY synchrony. Ca^2+^ level in AFD increases upon warming above a threshold temperature that is determined by cultivation temperature (Tc) in a manner dependent on guanylyl cyclases (GCYs) and TAX-2/4 CNG channels [93,94]; and decreases upon cooling. In well-fed animals, Ca^2+^ level in AIY increases synchronously with that in AFD when the ambient temperature is lower than the cultivation temperature (T < Tc) but decreases when T > Tc. Starvation disrupts the synchrony.

AFD-AIY synchrony is not only regulated in the well-fed-starved context but also within well-fed animals according to whether the current temperature is higher or lower than the past cultivation temperature. Our and other research groups recently identified molecules involved in this gating mechanism within AFD in well-fed animals, namely KIN-4 MAST kinase, MEC-2 stomatin, DGK-1 diacylglycerol kinase and PKC-1 protein kinase C [47,48] (Fig 9). It remains to be elucidated whether OLA-1 and ZYG-8 functionally interact with these factors, and how satiety information cross-talks with the comparison between current and memorized temperature in AFD.

In contrast to animals cultivated at 23°C, in animals cultivated at 17°C, the sites of OLA-1 action in promoting dispersion were in multiple neurons including thermotaxis neurons and BAG sensory neuron to promote dispersion (Fig 4). One possibility is that different neurons are involved in the transition from IT to dispersion according to cultivation temperature. Another possibility is that dispersion from cultivation temperature up or down a thermal gradient is asymmetric process, which is reminiscent of an argument that thermotaxis up or down a thermal gradient is asymmetric [63]. BAG is well-known to sense O_2_ [15] and CO_2_ [64–68] and either promotes or suppresses exploration in different contexts. When animals leave from a bacterial lawn along depletion of the food source, BAG promotes this leaving behavior [69]. In contrast, BAG suppresses leaving from a bacterial lawn before the exhaustion of the food source [70]. BAG also activates locomotion by decreasing fat storage [71]. It remains elusive whether OLA-1 regulates BAG activity and fat storage thereby promote dispersion from cultivation temperature, and whether OLA-1 and/or BAG functionally interact with the AWC-AIA axis that is important in disruption of thermotaxis toward colder temperature [30].

OLA-1 stands for Obg-like ATPase. Bacterial GTPase Obg induces multidrug tolerance [72]. OLA-1 is rather orthologous to YchF, another GTPase that belongs to YchF/YyaF subfamily of Obg-family [54]. Both bacterial YchF [73,74] and mammalian OLA1 [75,76] coordinate stress response by regulating translation. Since OLA-1 seems to act downstream of Ca^2+^ influx in AFD probably controlling transmitter release (Fig 6), OLA-1 might regulate expression level of molecules involved in exocytosis according to the feeding state.

We further showed that ZYG-8, an orthologue of human doublecortin-like kinase (DCLK), decelerates the transition from IT to dispersion of animals on a thermal gradient by acting upstream of or in parallel with OLA-1 in AFD (Fig 7). DCLKs stabilize microtubules (MTs) by binding to them via a conserved doublecortin (DCX) domain [77]. DCLKs regulates spindle assembly and cell fate determination during neurogenesis [78] and are associated with memory and cognitive functions and diseases such as Parkinson’s, Huntington’s, attention-deficit hyperactivity disorder (ADHD) and schizophrenia [79–81]. *C*. *elegans* ZYG-8 also interacts with MTs, promotes MT assembly in early embryo [50,82], organizes axonal MTs in post-mitotic neurons [83], and promotes axon regeneration after injury [84]. Given that local translation is important for axon regeneration [85,86], in which ZYG-8 and MTs are involved, and that OLA-1 can regulate translation, local translation of proteins involved in exocytosis via ZYG-8 and OLA-1 might be involved in the transition from IT to dispersion. Importantly, KIN-4 MAST kinase and MEC-2 Stomatin, which gate transmitter release from AFD [47], as well bind to MTs [87,88], implying a functional interaction between this gating mechanism and the starvation-mediating machinery involving OLA-1 and ZYG-8 (Fig 9).

## Materials and methods

### Experimental model and subject details

*C*. *elegans* strains were cultivated on nematode growth medium (NGM) plates seeded with *E*. *Coli* OP50 strain (Caenorhabditis Genetics Center (CGC), Twin Cities, MN, USA) as described [89]. N2 (Bristol) was used as the wild type strain unless otherwise indicated. Transgenic lines were generated by injecting plasmid DNA directly into hermaphrodite gonads as described [90]. Strains used in this study were listed in S1 Table.

### Behavioral assays

Population thermotaxis (TTX) assays were performed as described previously [52]. Briefly, 50 to 250 animals cultivated at 17°C or 23°C were placed on the center of assay plates without food with a temperature gradient of 17–23°C and were allowed to freely move for 1–24 h. The assay plate was divided into eight sections along the temperature gradient, and the number of adult animals in each section was scored. Ratio of animal numbers in each section was plotted in histograms. Thermotaxis indices were calculated as shown below:

$$\frac{\sum_{i=1}^{8}i∙N_{i}}{N}$$

N_i_: number of animals in each section i (i = 1 to 8), N: total number of animals on the test plate.

To prepare well-fed and starved animals, L4 animals were allowed to self-fertilize at 23°C for three days. Animals were washed two times with NG buffer (0.3% NaCl, 1 mM CaCl2, 1mM MgSO4, and 25 mM potassium phosphate, pH 6.0) and transferred to NGM plates with or without food, which were pre-incubated at 23°C, and incubated for two hours, respectively [31].

Pharyngeal pumping was analyzed as described previously [6]. Briefly, single well-fed or starved animals were transferred to an NGM plate with or without food with a picker. Two minutes later, pumping was scored visually for 30 seconds under MVX10 stereomicroscope (Olympus, Tokyo, Japan). Movement of animals in the Z-axis direction was restricted by mounting cover slips.

Locomotion rate was analyzed as described previously [7]. Briefly, single well-fed or starved animals were transferred to an NGM plate with or without food by mouth pipetting. Two minutes later, bending was counted visually for 20 seconds under MVX10 stereomicroscope (Olympus, Tokyo, Japan).

### Forward genetic screen for mutant animals that disperse slower from the cultivation temperature

For mutagenesis, wild type animals were treated with 47 mM ethyl methanesulfonate (EMS, Nacalai, Kyoto, Japan) for four hours at room temperature. F1 generation of the mutagenized animals were cultivated at 23°C for three days and were allowed to self-fertilize and to give rise to F2 progeny. F2 animals were then allowed to freely migrate on a thermal gradient without food for two hours, and animals that remained at the warmer regions of the thermal gradient were collected (S1A Fig). The collected animals were cultivated at 17°C overnight and again allowed to freely migrate on a thermal gradient without food for four hours. Animals that remained at the colder regions were collected and allowed to self-fertilize at 23°C. This selection cycle was repeated once again.

### Mapping of *nj80* mutation

Growth of *nj80* mutant animals was slow, and this slow growth was linked with the slow dispersion from cultivation temperature on a thermal gradient even after ten times of outcrossing with wild type N2 strain. We therefore used the slow growth phenotype to map *nj80* mutation. *nj80* animals were crossed with a wild-type polymorphic CB4856 strain, growth of which is comparable to N2, and F2 animals showing slow growth were isolated. Crossover sites were identified as described [91]. *nj80* mutation was mapped to a 1.2 Mb interval between 13.94 cM and 20.36 cM on linkage group *I*.

### Whole-genome sequencing

Genomic DNA was purified with Gentra Puregene Tissue Kit A (Qiagen, Hilden, Germany). The genome was sequenced in Advanced Genomics Center in National Institute of Genetics (Mishima, Japan).

### Plasmids

A DNA clone including *ola-1* cDNA (yk865b9) was provided by Dr. Yuji Kohara. A plasmid to express OLA-1::GFP was generated by ligating PCR genomic fragment containing 6 kb upstream sequences plus *ola-1* gene into SphI-AgeI site of pPD95.75. To generate plasmids to cell-specifically express *ola-1*, we fused promoter sequences of *unc-14*, *gcy-8*, *ceh-36*, *ttx-3*, *lin-11*, *glr-3*, *ncs-1*, *osm-6*, *glr-2*, *ets-5* or *avr-15*; the cDNA of *ola-1*; and the *unc-54* 3’UTR sequence by MultiSite Gateway Technology (Thermo Fisher Scientific, Waltham, MA, USA).

cDNA of *zyg-8* isoform b was cloned from DupLEX-A Yeast Two-Hybrid cDNA library *C*. *elegans* (Origene) into KpnI-NotI restriction sites of pIA139 (*snb-1p*::*VN173*). 5’ terminal sequence of *zyg-8* isoform a was cloned from N2 genome into *snb-1p*::*zyg-8b*. 6.3 kb upstream of the transcription start site of *zyg-8* isoform a, which partially includes *arx-3* gene, was amplified from N2 genome as *zyg-8* promoter sequence, and *snb-1* promoter of *snb-1p*::*zyg-8a* was replaced with *zyg-8* promoter. Details regarding the plasmid constructs can be obtained from the authors.

### Imaging

Expression of OLA-1::GFP and ZYG-8::GFP in head region were observed with BX53 upright microscope (Olympus, Tokyo, Japan). OLA-1::GFP expression in the whole body was observed with LSM880 confocal microscope (Zeiss).

Calcium imaging was performed as described elsewhere [37,41]. Briefly, a single adult animal that expressed genetically encoded calcium indicator GCaMP3 [59] and/or XCaMP-R [60] was placed on a 10% agar pad on a cover slip with 0.1 μm polystyrene beads (Polysciences, Warrington, PA, USA) and covered by another cover slip for immobilization [92]. The immobilized animals were placed on a Peltier-based temperature controller (Tokai Hit, Fujinomiya, Japan) on a stage of BX61WI microscope (Olympus, Tokyo, Japan). The red and green fluorescence was separated by the Dual-View optics system (Molecular Devices, Sunnyvale, CA, USA), and the images were captured by an EM-CCD camera C9100-13 ImageEM (Hamamatsu Photonics, Japan) at 1 frame per second. Excitation pulses were generated by SPECTRA light engine (Lumencor, Beaverton, OR, USA). The fluorescence intensities were measured by the MetaMorph imaging system (Molecular Devices).

### Analysis of simultaneous calcium imaging of AFD and AIY

The fluorescence intensities *F*(*t*) of XCaMP-R and GCaMP3 were rescaled as [*F*(*t*)−*F*_0_]/*F*_0_, where *F*_0_ is the minimum value of *F*(*t*) within an animal. For cross-correlation analysis, the rescaled AFD signals were detrended by subtracting their trends taken by Butterworth filter (passband edge frequency = 0.015 (Hz), stopband edge frequency = 0.03 (Hz), maximum loss in the passband = 1 (dB), maximum loss in the stopband = 30 (dB)), and the rescaled AIY signals were normalized by subtracting the means. We call these preprocessed AFD and AIY signals as *y*_AFD_(*t*) and *y*_AIY_(*t*), respectively. Cross-correlation function measuring the similarity between AFD and lagged AIY as a function of time lag was calculated as follows:

$${\tilde{C}}_{AFD,AIY}(m)=\left\{ \begin{array}{c} \sum_{n=0}^{N-m-1}y_{AFD}(n)y_{AIY}(n+m)\;(m\geq 0)\\ \sum_{n=0}^{N-|m|-1}y_{AIY}(n)y_{AFD}(n+|m|)\;(m<0) \end{array}\right.$$

$$C_{AFD,AIY}(m)={\tilde{C}}_{AFD,AIY}(m)/\left(N-|m|\right)$$

where m is time displacement (time lag), n is index of time point, and C_*AFD*,*AIY*_(m) is cross-correlation function. For Fourier analysis, *y*_AFD_ and *y*_AIY_ were windowed with Hamming window. To detect difference in AFD-AIY correlation strength between groups, we compared C_*AFD*,*AIY*_(0). To detect difference in time lag of AFD and AIY activities between groups, we compared time lags at which cross-correlation functions take their maxima.

In Fourier analysis, signal-to-noise ratio (SNR) of power spectra was calculated as follows:

$$SNR(f)=\frac{S(f)-{\bar{S}}_{noise}}{\sqrt{\frac{\sum_{n=1}^{N}{{(S}_{noise}(n)-{\bar{S}}_{noise})}^{2}}{N-1}}},$$

where f is frequency, S(f) is power of frequency, ${\bar{S}}_{noise}$ is mean of noise power, n is index for noise components (n = 1, …, N), and *S*_*noise*_ is noise power. The boundary between signal and noise was defined as follows: (1) The frequency components were sorted in descending order, (2) the cumulative sum of the power of the frequency components was taken, and (3) the point at which the cumulative sum exceeds 80% was defined as the boundary between signal and noise.

### Statistics

The error bars in histograms and line charts indicate the standard error of mean (SEM). In the boxplots, the bottom and top of boxes represent the first and third quartiles, and the band inside the box represents the median. The ends of the lower and upper whiskers represent the lowest datum still within the 1.5 interquartile range (IQR), which is equal to the difference between the third and the first quartiles, of the lower quartile, and the highest datum still within the 1.5 IQR of the upper quartile, respectively. For multiple-comparison, one-way analyses of variance (ANOVAs) were performed, followed by Dunnett or Tukey-Kramer tests, or Steel-Dwass test was performed. The Welch two-sample t-test or Wilcoxon rank sum test was used to compare two data sets. Statistical analyses were done by R programming language. When p-values were less than 0.05, 0.01 and 0.001, *, ** and *** were indicated, respectively.

## Supporting information

S1 Fig (A and B) Wild type and *ola-1(nj80)* animals were cultivated at 23°C for 3 days (A) or at 17°C for 5 days (B) and allowed to freely migrate on a thermal gradient for the time indicated. Number of animals at each section of the plate was scored. Fraction of animals (upper) and thermotaxis indices (lower) are shown. p values are indicated (Welch two-sample t-test at each time point). (C) A scheme of the screening. (D) Data of wild type and *ola-1(nj80)* mutant animals cultivated at 23°C and allowed to migrate on a thermal gradient for 1 hour from Figs 1A, 7A and S1A were put together. p values are indicated (Welch two-sample t-test). (E) Wild type and *ola-1(nj80)* animals were cultivated at 23°C for 3 days, put on a thermal gradient of which the central temperature was 23°C, and allowed to freely migrate for 1 hour.
(TIF)

S2 Fig Growth rate was compared among wild type, *ola-1(nj80)* and rescue strains. Eggs of each genotype were cultivated on NGM plates at 23°C for 55 hours (A) or at 17°C for 85 hours. Numbers of L4, nongravid adult and gravid adult were counted. p values for comparison between nongravid and gravid adults were indicated (Fisher’s exact test for count data with adjustment by Hochberg’s method).
(TIF)

S3 Fig (A-C) Wild type and *ola-1(nj80)* animals expressing GCaMP3 and tagRFP in AFD were cultivated at 23°C and allowed to freely migrate on a thermal gradient for 2 hours. Animals were then collected from sections 1 and 2, immobilized and subjected to Ca^2+^ imaging analysis with the indicated temperature stimulus warming from 15°C to 24°C with oscillation. The ratio of green to red fluorescence of each trial was normalized from zero to one, and the mean values of normalized ratio were plotted. Shadow represents the SEM. Data were collected from distinct animals. Temperature at which moving average of the normalized ratio change with 5 sec of window showed the maximum (B) and the half maximum for the first time (C) were plotted. n = 17, 20. p values were indicated (Wilcoxon rank sum test). (D) Fourier power spectrum of temperature and Ca^2+^ signals of AFD and AIY in Fig 6F. Data between 101 s and 400 s were analyzed. Dashed gray lines indicate 0.033 Hz that is the frequency of oscillatory warming stimuli. Black, red and blue curves indicate the mean values of the Fourier power spectrum of temperature, AFD and AIY, respectively. Light-colored curves indicate the individual data. (E-F) Signal to noise ratio of the Fourier power spectrum of Ca^2+^ signals of AFD (E) and AIY (F) at frequency of 0.033 Hz was plotted. n = 20, 25, 19, 17. p values were indicated (Steel-Dwass test).
(TIF)

S1 Table Strain list. (DOCX)
