# Supplementary figures and images for: OLA-1, an Obg-like ATPase, integrates hunger with temperature information in sensory neurons in C. elegans
Source: PLoS Genet. 2022 Jun 8;18(6):e1010219. doi: 10.1371/journal.pgen.1010219 (PMC9176836; doi:10.1371/journal.pgen.1010219)

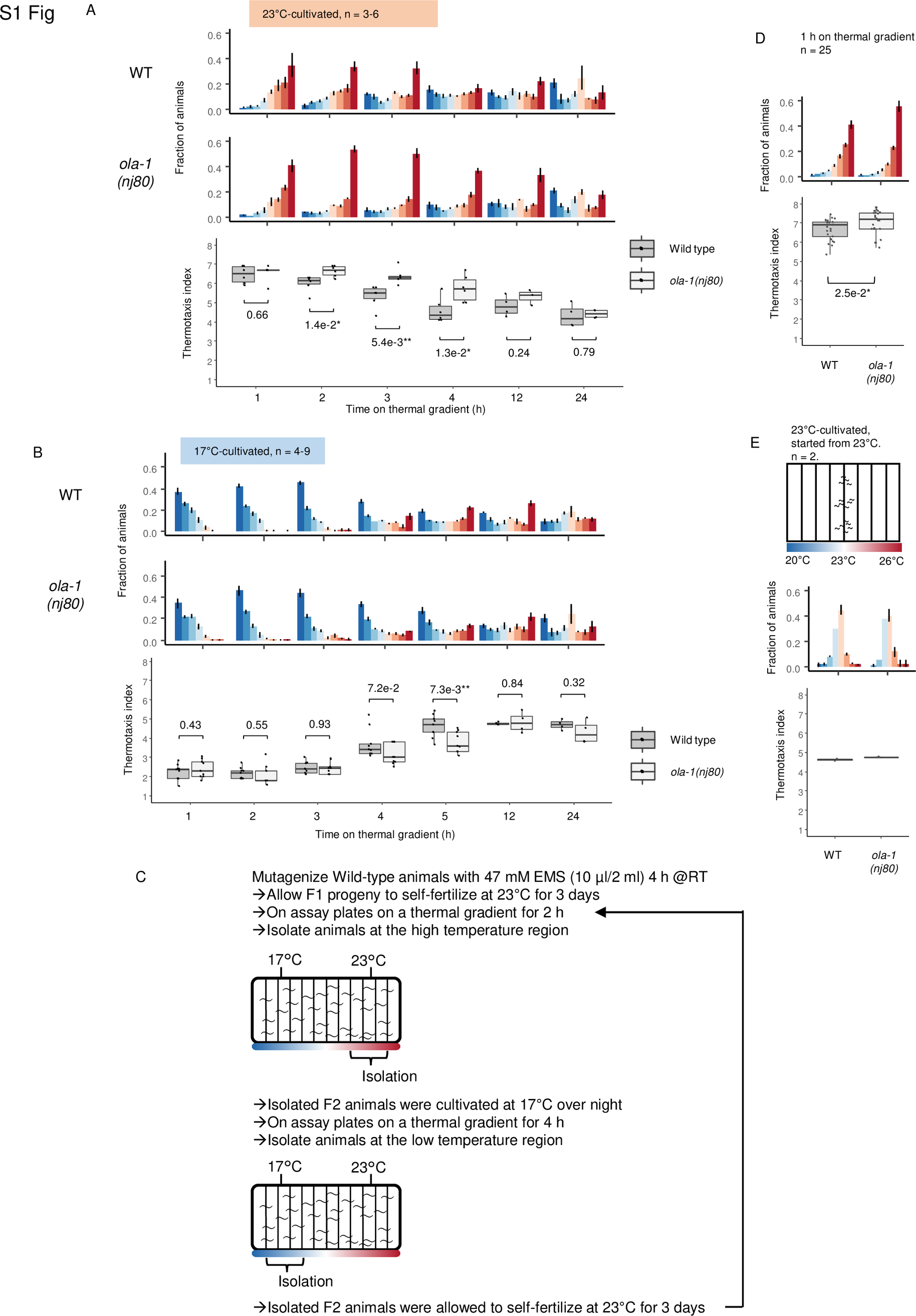

Supplement: S1 Fig — (A and B) Wild type and ola-1(nj80) animals were cultivated at 23°C for 3 days (A) or at 17°C for 5 days (B) and allowed to freely migrate on a thermal gradient for the time indicated. Number of animals at each section of the plate was scored. Fraction of animals (upper) and thermotaxis indices (lower) are shown. p values are indicated (Welch two-sample t-test at each time point). (C) A scheme of the screening. (D) Data of wild type and ola-1(nj80) mutant animals cultivated at 23°C and allowed to migrate on a thermal gradient for 1 hour from Figs 1A, 7A and S1A were put together. p values are indicated (Welch two-sample t-test). (E) Wild type and ola-1(nj80) animals were cultivated at 23°C for 3 days, put on a thermal gradient of which the central temperature was 23°C, and allowed to freely migrate for 1 hour. (TIF) [file pgen.1010219.s001.tif]

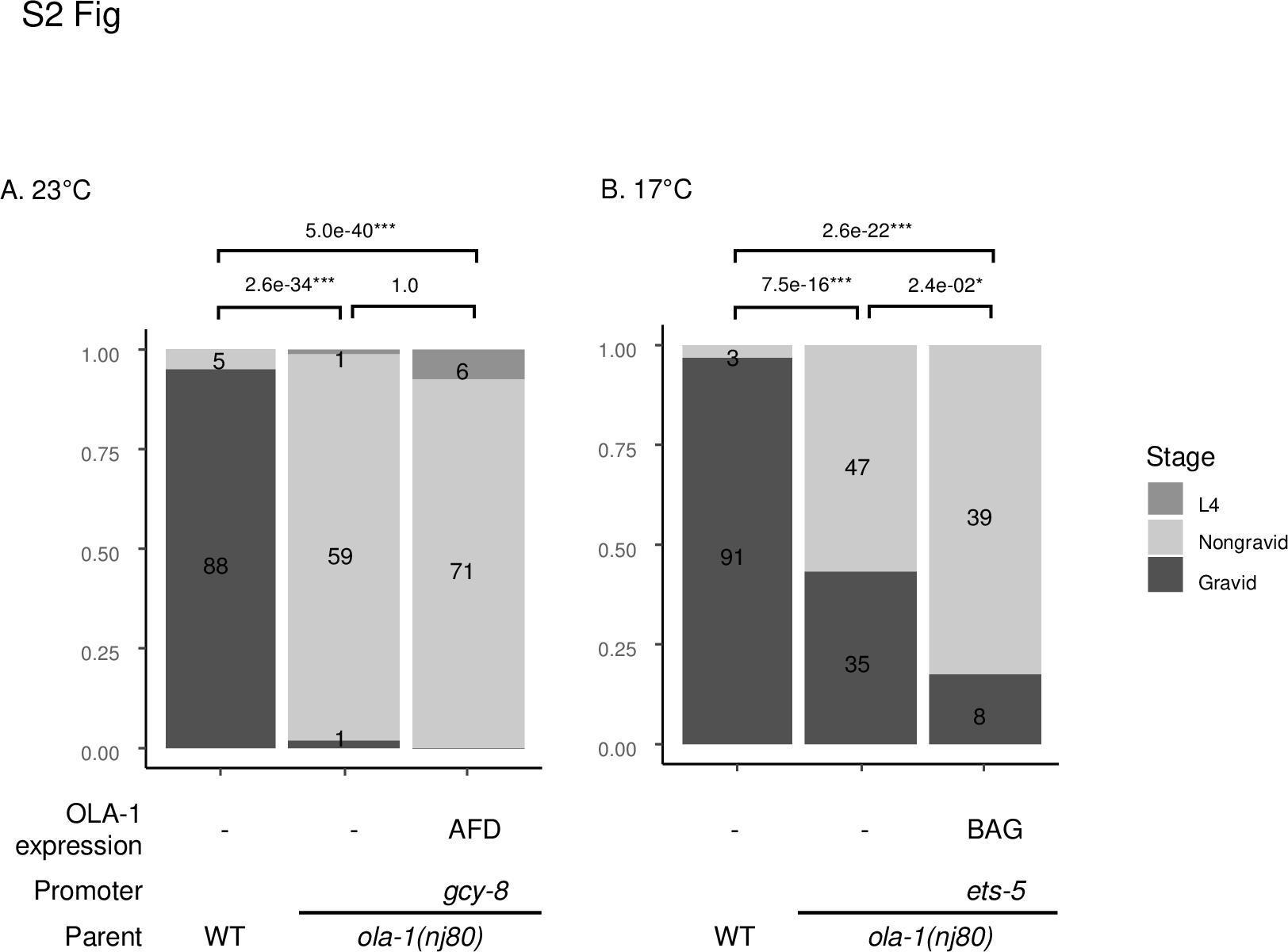

Supplement: S2 Fig — Eggs of each genotype were cultivated on NGM plates at 23°C for 55 hours (A) or at 17°C for 85 hours. Numbers of L4, nongravid adult and gravid adult were counted. p values for comparison between nongravid and gravid adults were indicated (Fisher’s exact test for count data with adjustment by Hochberg’s method). (TIF) [file pgen.1010219.s002.tif]

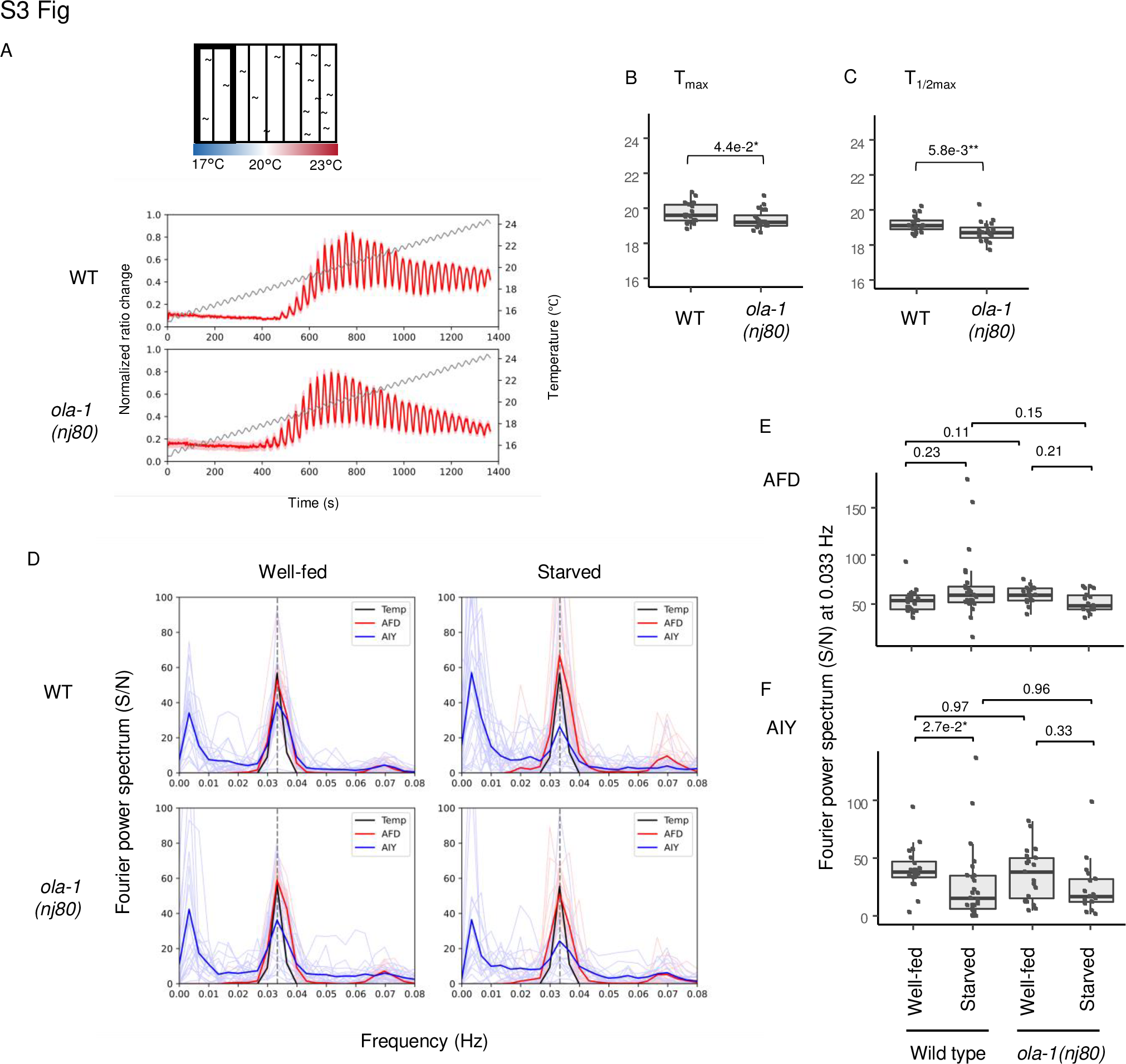

Supplement: S3 Fig — (A-C) Wild type and ola-1(nj80) animals expressing GCaMP3 and tagRFP in AFD were cultivated at 23°C and allowed to freely migrate on a thermal gradient for 2 hours. Animals were then collected from sections 1 and 2, immobilized and subjected to Ca2+ imaging analysis with the indicated temperature stimulus warming from 15°C to 24°C with oscillation. The ratio of green to red fluorescence of each trial was normalized from zero to one, and the mean values of normalized ratio were plotted. Shadow represents the SEM. Data were collected from distinct animals. Temperature at which moving average of the normalized ratio change with 5 sec of window showed the maximum (B) and the half maximum for the first time (C) were plotted. n = 17, 20. p values were indicated (Wilcoxon rank sum test). (D) Fourier power spectrum of temperature and Ca2+ signals of AFD and AIY in Fig 6F. Data between 101 s and 400 s were analyzed. Dashed gray lines indicate 0.033 Hz that is the frequency of oscillatory warming stimuli. Black, red and blue curves indicate the mean values of the Fourier power spectrum of temperature, AFD and AIY, respectively. Light-colored curves indicate the individual data. (E-F) Signal to noise ratio of the Fourier power spectrum of Ca2+ signals of AFD (E) and AIY (F) at frequency of 0.033 Hz was plotted. n = 20, 25, 19, 17. p values were indicated (Steel-Dwass test). (TIF) [file pgen.1010219.s003.tif]
