# Supplementary material for: OLA-1, an Obg-like ATPase, integrates hunger with temperature information in sensory neurons in C. elegans
Source: PLoS Genet. 2022 Jun 8;18(6):e1010219. doi: 10.1371/journal.pgen.1010219 (PMC9176836; doi:10.1371/journal.pgen.1010219)
Supplement: S1 Table — (DOCX) [file pgen.1010219.s004.docx]

## S4 Table. Strain list.

| **Strain ID** | **Genotype** |
| --- | --- |
| N2 | wild type |
| IK0940 | *ola-1(nj80)* |
| IK3059 | *ola-1(nj80); njEx1250[unc-14p::ola-1a(+), ges-1p::EGFP]* |
| IK3107 | *ola-1(nj80); njEx1283[gcy-8p::ola-1a(+), ges-1p::EGFP]* |
| IK3197 | *ola-1(nj80); njEx1346[osm-6p::ola-1a(+), ges-1p::EGFP]* |
| IK3202 | *ola-1(nj80); njEx1351[avr-15p::ola-1a(+), ges-1p::EGFP]* |
| IK3313 | *ola-1(nj80); njEx1377[ets-5p::ola-1a(+), ges-1p::EGFP]* |
| IK3378 | *ola-1(nj80); njEx1419[ncs-1p::ola-1a(+), ges-1p::EGFP]* |
| IK3381 | *ola-1(nj80); njEx1421[gcy-8p::ola-1a(+), ceh-36p::ola-1a(+), ttx-3p::ola-1a(+), lin-11p::ola-1a(+), glr-3p::ola-1a(+), ges-1p::EGFP]* |
| IK3383 | *ola-1(nj80); njEx1423[ges-1p::ola-1a(+), ges-1p::EGFP]* |
| IK3384 | *ola-1(nj80); njEx1424[ceh-36p::ola-1a(+), ges-1p::EGFP]* |
| IK3385 | *ola-1(nj80); njEx1425[glr-2p::ola-1a(+), ges-1p::EGFP]* |
| IK1974 | *njIs34[gcy-8p::GCaMP3, gcy-8p::TagRFP]* |
| IK3122 | *ola-1(nj80); njIs34[gcy-8p::GCaMP3, gcy-8p::TagRFP]* |
| IK3461 | *zyg-8(b235ts)* |
| IK3478 | *ola-1(nj80); zyg-8(b235ts)* |
| IK3532 | *zyg-8(b235); njEx1508[zyg-8p::zyg-8a cDNA 10 ng/ul, ges-1p::EGFP 90 ng/ul] #1* |
| IK3533 | *zyg-8(b235); njEx1508[zyg-8p::zyg-8a cDNA 10 ng/ul, ges-1p::EGFP 90 ng/ul] #2* |
| IK3828 | *zyg-8(b235); njEx1659[gcy-8p::zyg-8a cDNA, ges-1p::GFP]* |
| IK3837 | *ola-1(nj80); njEx1668[ola-1p::ola-1::GFP]* |
| IK3865 | *zyg-8(b235); njEx1680[zyg-8p::zyg-8a cDNA::GFP, ges-1p::tagRFP]* |
| IK4055 | *njEx1775[gcy-8Lp::XCaMP-R::unc-54 3’UTR, AIYp::GCaMP-3]* |
| IK4087 | *ola-1(nj80); njEx1775[gcy-8Lp::XCaMP-R::unc-54 3’UTR, AIYp::GCaMP-3]* |
|  |  |
